# Genome-wide identification and expression analysis of the cucumber *PP2C* gene family

**DOI:** 10.1186/s12864-022-08734-y

**Published:** 2022-08-06

**Authors:** Guobin Zhang, Zeyu Zhang, Shilei Luo, Xia Li, Jian Lyu, Zeci Liu, Zilong Wan, Jihua Yu

**Affiliations:** 1 State Key Laboratory of Aridland Crop Science, Gansu Agricultural University, Lanzhou, 730070 China; 2 College of Horticulture, Gansu Agricultural University, Lanzhou, 730070 China; 3 Gansu Institute of Geological and Natural Disaster Prevention, Lanzhou, 730000 China

**Keywords:** Cucumber, Protein phosphatase 2C, Stress response

## Abstract

**Background:**

Type 2C protein phosphatase (PP2C) is a negative regulator of ABA signaling pathway, which plays important roles in stress signal transduction in plants. However, little research on the *PP2C* genes family of cucumber (*Cucumis sativus* L.), as an important economic vegetable, has been conducted.

**Results:**

This study conducted a genome-wide investigation of the *CsPP2C* gene family. Through bioinformatics analysis, 56 *CsPP2C* genes were identified in cucumber. Based on phylogenetic analysis, the *PP2C* genes of cucumber and *Arabidopsis* were divided into 13 groups. Gene structure and conserved motif analysis showed that *CsPP2C* genes in the same group had similar gene structure and conserved domains. Collinearity analysis showed that segmental duplication events played a key role in the expansion of the cucumber *PP2C* genes family. In addition, the expression of *CsPP2Cs* under different abiotic treatments was analyzed by qRT-PCR. The results reveal that *CsPP2C* family genes showed different expression patterns under ABA, drought, salt, and cold treatment, and that *CsPP2C3*, *11*–*17*, *23*, *45*, *54* and *55* responded significantly to the four stresses. By predicting the cis-elements in the promoter, we found that all *CsPP2C* members contained ABA response elements and drought response elements. Additionally, the expression patterns of *CsPP2C* genes were specific in different tissues.

**Conclusions:**

The results of this study provide a reference for the genome-wide identification of the *PP2C* gene family in other species and provide a basis for future studies on the function of *PP2C* genes in cucumber.

**Supplementary Information:**

The online version contains supplementary material available at 10.1186/s12864-022-08734-y.

## Background

Plants have developed multiple response mechanisms in response to salt, drought and cold stress, among which the regulation of related gene expression and protein modification are two important pathways. Protein reversible phosphorylation, a protein modification process that regulates multiple physiological responses in plants, is catalyzed by protein kinases (PKs) and phosphatases (PPs) [1, 2]. PKs primarily phosphorylate serine (Ser), threonine (Thr) and tyrosine (Tyr), while PPs can reverse this function by eliminating phosphate groups [3]. Based on substrate specificity, protein phosphatases (PPs) can be divided into three groups: Ser/Thr phosphatases (STPs), protein Tyr phosphatases (PTPs), and dual-specificity phosphatases (DSPTPs). Moreover, PTPs are further classified into phosphoprotein metal phosphatases (PPM) and phosphoprotein phosphatases (PPP) based on crystal structure, amino acid sequence and specific response to inhibitors [4]. The PPP family contains a variety of protein phosphatases, including PP1, PP4, PP5, PP6, PP7, PP2A and PP2B [5, 6]. At present, very little is known about the PPP family. However, we know that PP2A changes cell morphology in the PPP family during elongation, affecting root growth and root hair growth [7]. PP1 is a highly conserved and ubiquitous phosphatase in eukaryotes [8]. Nine members of the PP1 gene family have been identified in *Arabidopsis* [9, 10]. In addition, a PP1 gene up-regulated by biological stress was found in *Phaseolus vulgaris* [11].

Type 2C protein phosphatases (PP2C) are Mg^2+^/Mn^2+^ dependent serine/threonine phosphatases that are closely related to the PPP family, but have no sequence homology, thus forming a unique group belonging to the PPM family. The *PP2C* gene family is evolutionarily conserved, significantly regulates stress signaling pathways, and is primarily present in bacteria, fungi, plants and animals [12]. Plant PP2C family proteins have unique structures that contain a conserved catalytic region at the N- or C-terminus and an unconserved extension region of varying length at the other end [13]. The diversity of their structures indicates that PP2C family proteins have different functions in the signal transduction mechanism [14, 15]. A total of 80 *PP2C* genes were identified in *Arabidopsis*, which were divided into 13 subsets (A-J) according to their evolutionary relationships. Six of the nine members of subgroup A (ABI1, ABI2, AHG1, AHG3/ ATPP2CA, HAB1 and HAB2) negatively regulate ABA signaling, and the remaining three members (HAI1, HAI2/AIP1 and HAI3) respond differently to stress than the other six members [16–18]. PP2C of subgroup A inactivated PP2C through dephosphorylation, inhibited ABA receptor (PYR/PYL/RCRA) activity, and negatively regulated ABA signaling [19]. The identification of two PP2C mutants, *abi1* and *abi2*, provides favorable support for their negative regulatory function of ABA signaling [20]. Group B participated in the mitogen-activated protein kinase (MAPK) signaling pathway and catalyzed the dephosphorylation of MAPKs, leading to their inactivation [21, 22]. Group C is involved in the regulation of flower development [23]. Group D members can respond to salt and alkali stress [24]. Group E *PP2C* is involved in the regulation of stomatal signaling, for example, as a response to dehydration, PP2C interacts with MPK3 and MPK6 to induce stomatal closure and inhibit water loss due to transpiration [25, 26]. Group F *PP2C* is involved in the induction of bacterial stress response [27]. Currently, the *PP2C* gene family has been identified and studied in a variety of plants other than *Arabidopsis*, such as rice [28], maize [29], and *Brachypodium distachyon* [12]. Rice *OsPP108* belonging to subgroup A could be highly expressed under ABA, drought, and salt stress; when overexpressed in *Arabidopsis*, transgenic *Arabidopsis* showed stronger stress resistance [30]. Overexpression of the maize *ZmPP2C* gene in *Arabidopsis* reduced the sensitivity to ABA and the tolerance to drought and salt treatment [31]. *FaABI1* in strawberry had negative regulatory effects on fruit ripening [32]. The expression level of the *PP2C* gene regulated by endogenous ABA in *Arabidopsis* was significantly up-regulated under exogenous ABA and stress [19]. In conclusion, the *PP2C* gene family plays a critical role in plant development and environmental stress.

Although the *PP2C* gene family has been extensively studied in other species, there are few reports on the *PP2C* gene family in cucumber. Therefore, identifying the *PP2C* gene family in cucumber and analyze its expression under stress is of great significance. In this study, 56 *PP2C* genes were identified at the genome-wide level in cucumber by bioinformatics methods. The physicochemical properties, protein secondary structure, chromosome location, phylogenetic analysis, gene structure, conserved motifs and cis-acting elements, and relative expression of 56 *PP2C* genes were systematically analyzed, providing reference for the function of abundant *PP2C* gene families. This study will lay the foundation for understanding the potential molecular mechanism of PP2C in stress signal transduction.

## Results

### Identification and basic information of the *CsPP2C* gene family

In this study, we determined that 56 putative *CsPP2C* were present in the cucumber genome through BLASTp using 80 *AtPP2C* protein sequences as references. From the analysis of their physical and chemical properties (Table 1), the 56 *CsPP2C* genes identified encode proteins varying from 233 to 813 amino acids in length, isoelectric point (PI) value ranging from 4.5 to 9.61, and the molecular weight ranging from 30 kDa to 90 kDa. The total average hydrophobic index of the 56 *CsPP2C* gene family members were all less than zero and therefore encoded hydrophilic proteins. The subcellular localization prediction indicated that most of the CsPP2C proteins might be located in the nucleus, chloroplast, or cytoplasm, while only *CsPP2C48* might be located in the endoplasmic reticulum and *CsPP2C50* in the cytoskeleton.

**Table 1:** List of 56 *CsPP2C* genes and their basic characterizations

Gene identifier	Gene name	Size (aa)	Mass (kDa)	pI	Instability index	Aliphatic index	Grand average of hydropathicity	Subcellular localization
CsaV3_1G004080.1	CsPP2C1	348	37.934	7.67	28.63	89.97	−0.299	Nuclear
CsaV3_1G034580.1	CsPP2C2	426	46.884	5.06	57.49	73.66	−0.45	Nuclear
CsaV3_1G035680.1	CsPP2C3	358	39.549	5.26	69.22	72.4	−0.499	Chloroplast
CsaV3_1G036330.1	CsPP2C4	392	42.073	5.97	52.12	83.93	−0.194	Chloroplast
CsaV3_1G038430.1	CsPP2C5	370	41.493	8.01	38.05	84.84	−0.254	Nuclear
CsaV3_1G038460.1	CsPP2C6	428	46.271	7.96	32.07	87.01	−0.173	Chloroplast
CsaV3_1G039750.1	CsPP2C7	380	41.393	5.24	54.5	78.47	−0.323	Chloroplast
CsaV3_2G003570.1	CsPP2C8	357	39.617	5.27	48.44	76.78	−0.341	Nuclear
CsaV3_2G006810.1	CsPP2C9	367	40.752	6.08	40.34	78.88	−0.283	Nuclear
CsaV3_2G010540.1	CsPP2C10	484	53.551	5.74	42.41	74.32	−0.508	Chloroplast
CsaV3_2G012660.1	CsPP2C11	275	31.235	9.51	43.29	76.22	−0.471	Chloroplast
CsaV3_2G016210.1	CsPP2C12	397	44.019	8.96	46.3	87.41	−0.313	Chloroplast
CsaV3_2G024970.1	CsPP2C13	424	46.836	8.25	53.61	85.94	−0.302	Cytoplasmic
CsaV3_2G033210.1	CsPP2C14	309	34.579	6.29	46.91	82.33	−0.384	Cytoplasmic
CsaV3_3G000550.1	CsPP2C15	390	42.936	7.7	46.19	90.44	−0.219	Nuclear
CsaV3_3G001890.1	CsPP2C16	813	89.039	5.29	46.54	76.17	−0.473	Nuclear
CsaV3_3G003600.1	CsPP2C17	523	57.737	5.39	50.39	75.3	−0.409	Nuclear
CsaV3_3G013890.1	CsPP2C18	414	45.372	5.27	33.49	84.69	−0.282	Chloroplast
CsaV3_3G014600.1	CsPP2C19	521	56.883	5.31	42.17	89.08	−0.227	Nuclear
CsaV3_3G016530.1	CsPP2C20	421	45.192	5.61	68.05	74.8	−0.302	Nuclear
CsaV3_3G019720.1	CsPP2C21	387	42.372	5.39	42	90.03	−0.105	Chloroplast
CsaV3_3G022030.1	CsPP2C22	349	38.668	5.54	36.64	78.51	−0.406	Chloroplast
CsaV3_3G027970.1	CsPP2C23	233	26.258	6.76	36.69	81.2	−0.488	Chloroplast
CsaV3_3G035300.1	CsPP2C24	370	41.113	8.84	39.84	91.68	−0.326	Nuclear
CsaV3_3G038810.1	CsPP2C25	390	43.875	7.18	7.18	88.46	−0.299	Chloroplast
CsaV3_3G043720.1	CsPP2C26	424	46.135	5.61	41.4	91.46	−0.119	Chloroplast
CsaV3_3G047510.1	CsPP2C27	281	31.084	6.51	36.8	88.86	−0.351	Chloroplast
CsaV3_4G009460.1	CsPP2C28	236	26.175	6.07	40.16	87.63	−0.453	Cytoplasmic
CsaV3_4G025000.1	CsPP2C29	712	79.584	5.76	40.03	72.46	−0.574	Nuclear
CsaV3_4G026720.1	CsPP2C30	446	49.889	8.55	39.04	71.41	−0.591	Nuclear
CsaV3_4G033570.1	CsPP2C31	283	31.385	5.88	37.1	81.98	−0.398	Cytoplasmic
CsaV3_4G034220.1	CsPP2C32	428	46.643	5.49	48.53	79.25	−0.303	Chloroplast
CsaV3_4G035500.1	CsPP2C33	325	35.755	5.32	44.62	86.4	−0.26	Nuclear
CsaV3_4G036320.1	CsPP2C34	293	31.576	5.07	43.56	78.26	−0.313	Cytoplasmic
CsaV3_4G036470.1	CsPP2C35	364	39.767	5.22	33.4	73.46	−0.374	Chloroplast
CsaV3_4G037450.1	CsPP2C36	386	41.981	5.31	54.25	82.9	−0.199	Nuclear
CsaV3_5G006460.1	CsPP2C37	389	43.513	7.24	42.56	93.7	−0.23	Chloroplast
CsaV3_5G010270.1	CsPP2C38	363	39.279	6.68	58.25	80.83	−0.269	Chloroplast
CsaV3_5G034510.1	CsPP2C39	433	46.590	8.64	39.69	85.36	−0.194	Chloroplast
CsaV3_6G000080.1	CsPP2C40	372	40.917	5.27	54.21	91.48	−0.177	Cytoplasmic
CsaV3_6G001340.1	CsPP2C41	402	44.641	5.91	61.87	73.71	−0.533	Chloroplast
CsaV3_6G003780.1	CsPP2C42	400	44.337	8.83	32.12	78	−0.416	Cytoplasmic
CsaV3_6G005520.1	CsPP2C43	403	44.260	4.5	31.29	83.4	−0.268	Cytoplasmic
CsaV3_6G016880.1	CsPP2C44	715	79.492	5.73	37.16	82.64	−0.473	Nuclear
CsaV3_6G022710.1	CsPP2C45	349	38.860	8.59	42.43	77.59	−0.406	Nuclear
CsaV3_6G028560.1	CsPP2C46	377	42.560	6.34	41.69	89.2	−0.294	Cytoplasmic
CsaV3_6G031110.1	CsPP2C47	275	30.192	5.24	36.02	81.93	−0.268	Chloroplast
CsaV3_6G032130.1	CsPP2C48	553	59.388	4.7	48.54	85.17	−0.158	Endoplasmic reticulum
CsaV3_6G047490.1	CsPP2C49	398	44.604	7.27	53.52	88.87	−0.24	Chloroplast
CsaV3_6G052400.1	CsPP2C50	275	30.748	4.9	51.66	84.73	−0.446	Cytoskeleton
CsaV3_7G001180.1	CsPP2C51	287	31.871	9.61	46.22	97.42	−0.137	Chloroplast
CsaV3_7G004290.1	CsPP2C52	471	51.927	5.12	50.33	69.94	−0.417	Chloroplast
CsaV3_7G005840.1	CsPP2C53	291	31.923	8.76	30.42	90.1	−0.331	Cytoplasmic
CsaV3_7G007970.1	CsPP2C54	382	42.507	8.51	47.81	89.06	−0.277	Chloroplast
CsaV3_7G030300.1	CsPP2C55	393	43.077	4.64	52.99	89.52	−0.161	Cytoplasmic
CsaV3_7G031840.1	CsPP2C56	367	40.317	5.05	38.99	68.88	−0.496	Chloroplast

### Chromosome distribution and collinearity analysis of the *PP2C* gene family in cucumber

**Fig. 1:**
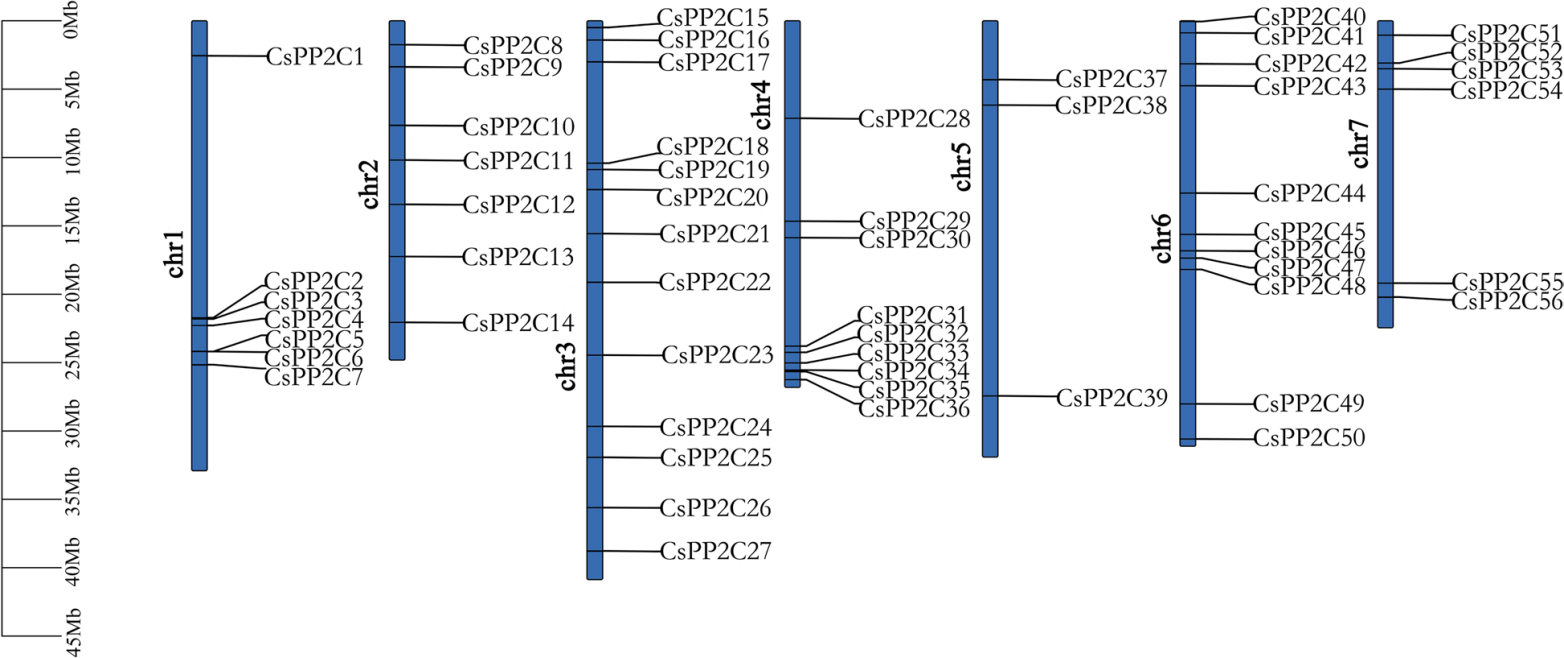
Chromosomal distribution and localization of *CsPP2Cs*. The chromosome names are shown at the left of each chromosome. The chromosome scale is in millions of bases (Mb) on the left

**Fig. 2:**
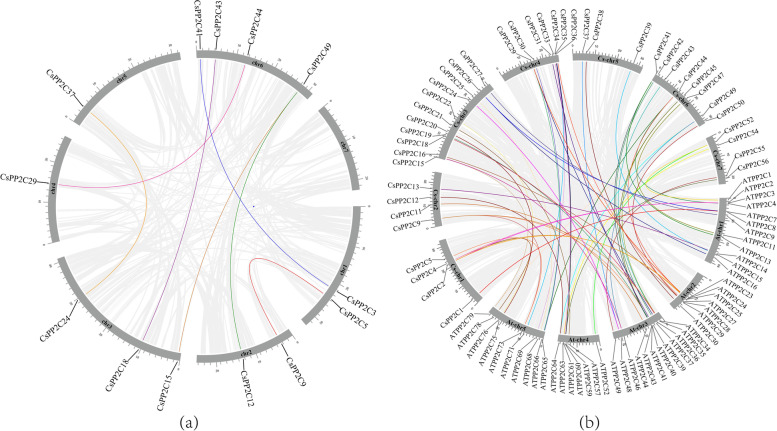
Collinearity analysis of the *PP2C* gene family in cucumber. **a** Chromosomes 1–7 are represented by gray rectangles. The gray lines indicate synteny blocks in the cucumber genome, while the lines of different colors between chromosomes delineate segmental duplicated gene pairs. **b** Synteny analysis of *PP2Cs* between cucumber and *Arabidopsis*. Gray lines denote the collinear blocks between cucumber and *Arabidopsis* genomes and the lines of different colors denote the syntenic gene pairs of *PP2Cs*. Gray rectangles represent respectively the cucumber chromosomes (1–7) and *Arabidopsis* chromosomes (1–5)

To obtain information on the position of *CsPP2C* genes on the chromosome, we used TBtools to map the chromosomal location (Fig. 1). A total of 56 *PP2C* genes were anchored to corresponding chromosomes and designated as *CsPP2C1*–*CsPP2C56* according to their order on the chromosomes, among which chromosomes 3 and 6 were more distributed, and chromosome 5 was the least distributed, with only 3 *PP2C* genes. Closely related genes located within a distance of less than 200 kb on the same chromosome were defined as tandem duplications, otherwise they were defined as segmental duplications [33]. To further understand the expansion mechanism of the *CsPP2Cs*, we examined segmental and tandem duplications within the cucumber genome. Our results showed that the *PP2C* gene family had no tandem duplication gene pairs, but that there were seven fragment repeat gene pairs (Fig. 2 a). In the seven pairs of collinear relationships, *CsPP2C49* was paired with *CsPP2C15* and *CsPP2C12*, respectively, while the others were one-to-one paired.

In addition, we also detected homologous *PP2C* gene pairs between cucumber and *Arabidopsis*. There were 59 collinear gene pairs between 41 *CsPP2Cs* and 48 *AtPP2Cs* (Fig. 2 b). The maximum number of homologous genes in cucumber was 11 pairs on chromosome 3, while the minimum number was three pairs on chromosome 5. According to this result, we speculated that cucumber and *Arabidopsis* may have high homology.

### Analysis of d_N_/d_s_ values between collinear gene pairs

To further investigate the divergence and selection in duplication of *PP2C* genes, the non-synonymous substitution rate (d_N_), synonymous substitution rate (ds), and d_N_/d_s_ values were evaluated for the homologous gene pairs among cucumber and *Arabidopsis* (Table S1). Where d_N_/d_S_ > 1 is the positive selection, d_N_/d_S_ = 1 is the neutral selection, and 0 < d_N_/d_S_ < 1 is the purifying selection [34]. The d_N_/d_s_ value of all cucumber gene pairs was less than 1. Similarly, the d_N_/d_s_ value of all collinear gene pairs in cucumber and *Arabidopsis* was less than 1. These data suggest that these genes were primarily under purifying selection during evolution and could help to maintain the basic function of this gene.

### Phylogenetic analysis of *CsPP2C* genes

**Fig. 3:**
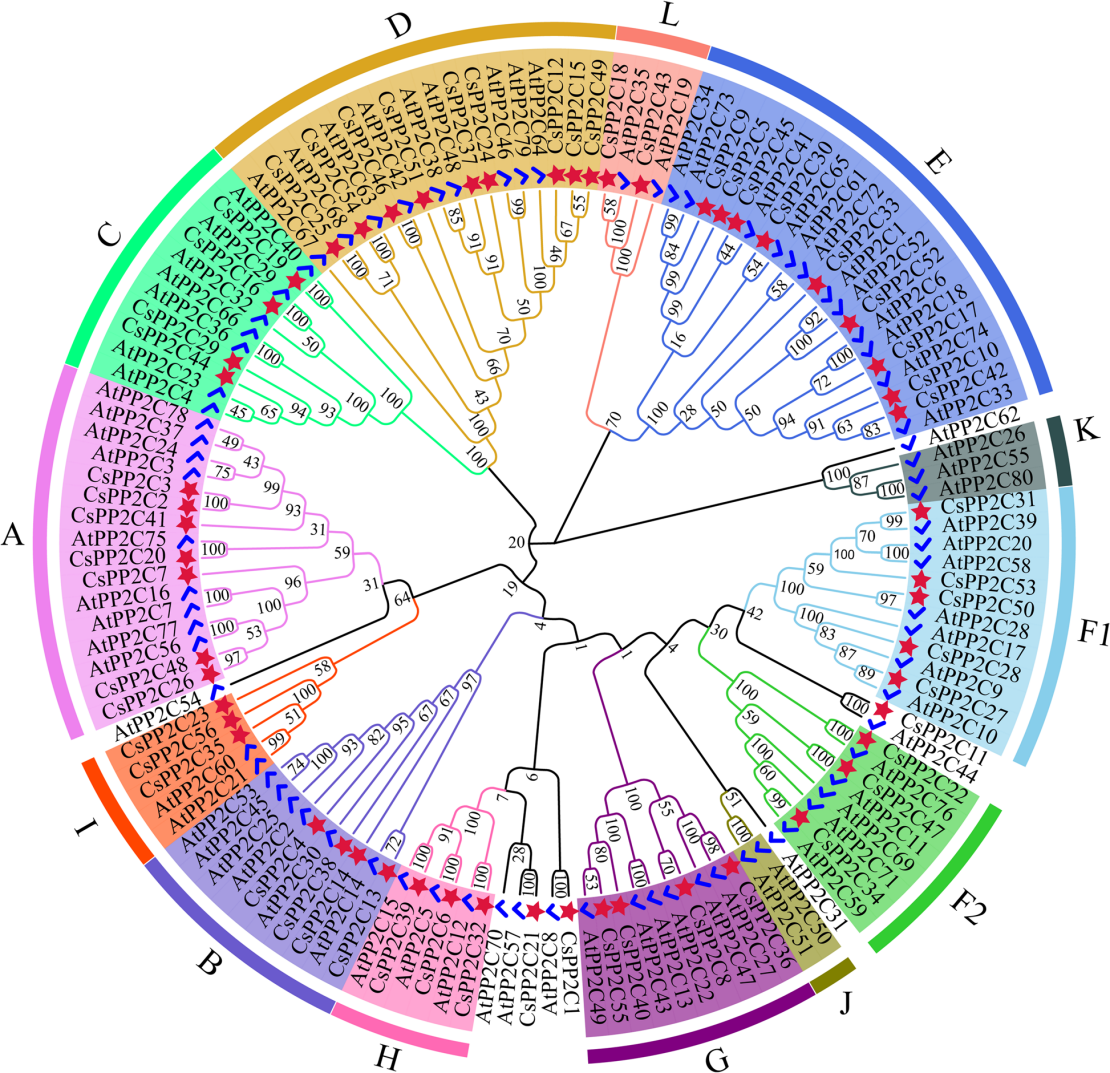
Phylogenetic analysis of *PP2C* proteins among cucumber and *Arabidopsis*, red asterisks represent *CsPP2C*, black circles represent *Arabidopsis.* The phylogenetic tree was constructed by MEGA 7 using the Maximum Likelihood Method (1000 bootstrap)

To investigate the phylogenetic relationships of *PP2C* genes between cucumber and *Arabidopsis*, we used the maximum likelihood method to construct a phylogenetic tree based on 80 *PP2C* genes in *Arabidopsis* and 56 in cucumber (Fig. 3). The phylogenetic analyses indicated that each subfamily includes PP2C proteins from cucumber and *Arabidopsis*, and that the genes of cucumber and *Arabidopsis* tended to form independent branches in each subgroup, that is, cucumber genes clustered together, and *Arabidopsis* genes clustered together. The 56 CsPP2C proteins were divided into 13 subgroups (A-L), while *CsPP2C1*, *21*, and *11* were not clustered with any other group. This was similar to the grouping of *PP2C* in *Arabidopsis* and rice. Each group included seven, four, four, nine, nine, five, three, four, three, three, zero, zero, and two *CsPP2C* genes. Aside from subgroup J and K (only the *AtPP2C* gene), the distribution of *PP2Cs* in cucumber and *Arabidopsis* subgroups was similar. This suggests that the *PP2C* gene family may have evolved from a common ancestor.

### Gene structural and conserved domain analyses of *CsPP2Cs*

**Fig. 4:**
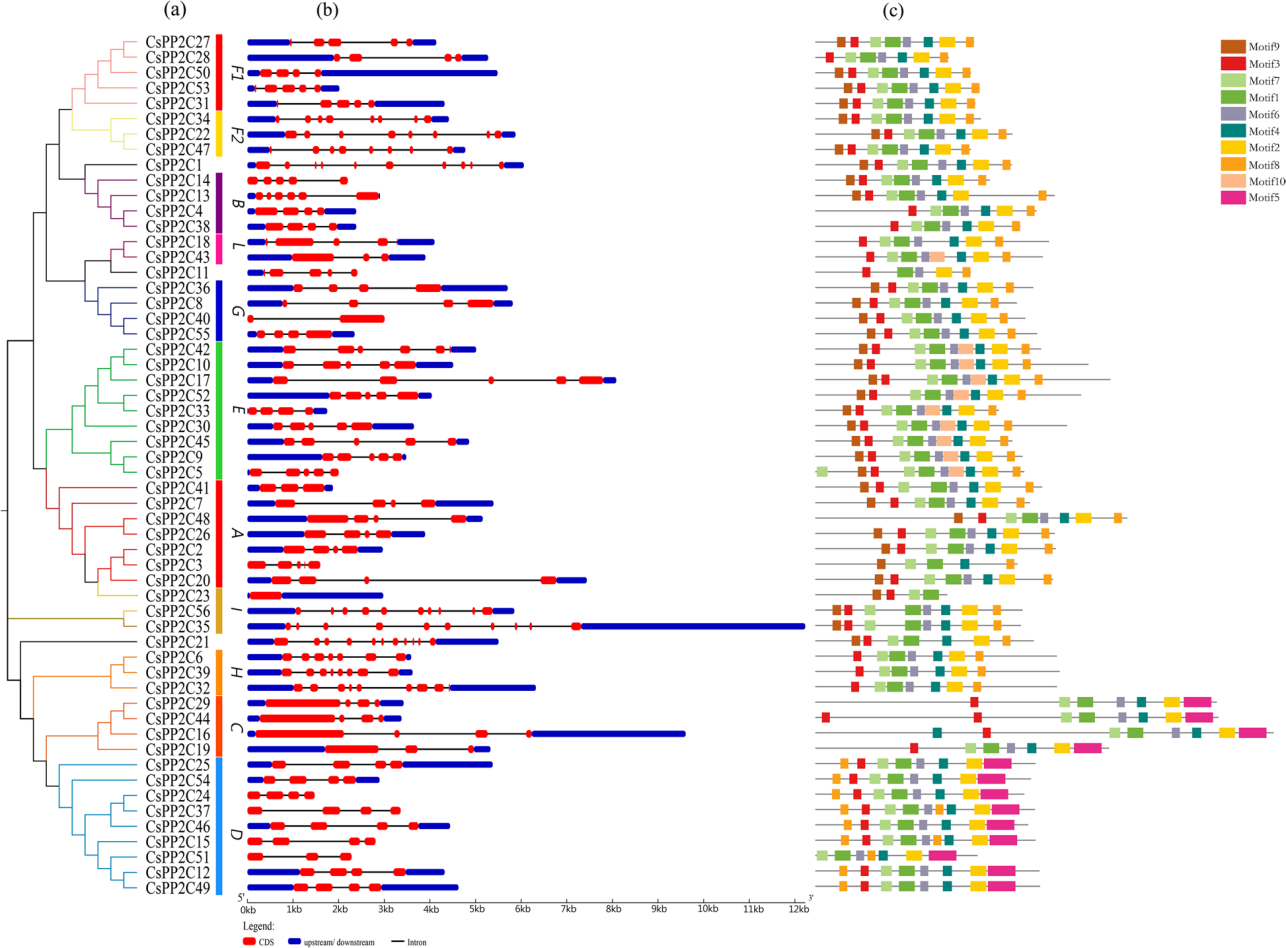
Phylogenetic tree, gene structure and motif analysis of *CsPP2Cs*. **a** The phylogenetic tree of *CsPP2C* is divided into ten groups. **b** Exon–intron structures of *CsPP2C* genes. **c** Distribution of all motifs identified by MEME

Since the pattern diversity of exon/intron structure and protein domain plays an important role in the evolution of gene families, we studied the exon/intron structure patterns of *CsPP2C* genes and conserved domain based on their phylogenetic relationships (Fig. 4 a). Studies on exon/intron structure showed that most members of the same subfamily have similar exon/intron numbers but differ in length (Fig. 4 b). The structure of the *CsPP2C* gene in each group was mostly similar, but there were differences in the exon/intron arrangement of some genes. For example, in group F, the 3′-end of *CsPP2C50* has the longest non-coding region and the number of exons in group F2 (8 exons) was almost twice that in group F1 (4–5 exons). In addition, *CsPP2C35* in group I has 10 exons, and its longest gene fragment is more than 12 kb long. *CsPP2C40* in group G had no non-coding regions, and only two exons, the lowest number of exons in all *PP2C* genes. *CsPP2C14*, *40*, 3, *24*, *37*, *15*, and *51* have no upstream and downstream gene sequences, and most were located in group D. This indicated that the *CsPP2C* gene was relatively conservative in its evolution, ensuring the integrity of its gene structure so that there is little change in its function.

To identify common motifs among different groups of *CsPP2C* proteins, we used the MEME motif search tool to identify 10 conserved motifs (Table 2). As shown in Fig. 4. c, proteins in the same group exhibited similar motif distribution patterns. Motifs 1, 2 (except *CsPP2C3*, *23*), 3 (except *CsPP2C51*), 4, 6, and 7 were found in all *CsPP2C* genes. In addition to the common motif, there were specific motifs in each group. For example, motif 8 was not present in group C, but was present in all other groups. Motif 5 was present in group C and D, but not in the other groups. Motif 9 was not found in group C, D, and H, while it was found in all other groups. According to these results, the *CsPP2C* genes in the same subgroup had similar conserved motif composition and distribution, suggesting that the *CsPP2C* members in the same cluster likely share similar functions.

**Table 2:** Conserved motifs in the amino acid sequences of *CsPP2C* proteins

Motif	Width Multilevel	consensus sequence
1	29	SGSTALVALIQGDTLYVANVGDSRAVLAR
2	29	LTPEDEFLILASDGLWDVLSNZEAVDIVR
3	15	AFFGVFDGHGGPGAA
4	17	GGLAVSRAIGDFYLKQY
5	50	PRNGSAKRLVKAALQEAAKKREMRYSDLKKIDRGVRRHFHDDITVIVVFL
6	15	AIQLSVDHKPSREDE
7	20	WEKAJKKAFLKTDEEFLSLV
8	15	RGSKDBISVIVVQFK
9	16	QGKRGEMEDAHIVWED
10	27	AERIKQCKGRVFALQDEPEVYRVWLPN

### Cis-element analysis of the *CsPP2Cs* promoter in cucumber

**Fig. 5:**
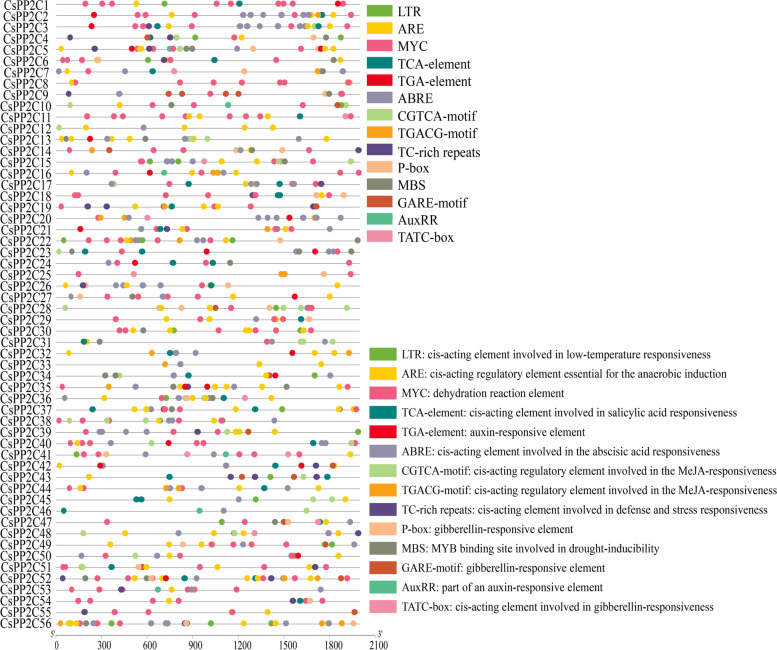
Putative cis-elements existed in the 2 kb upstream region of cucumber *PP2C* genes. The elements which respond to hormones are displayed in differently coloured boxes. The homeopathic elements represented by different color boxes and their names and functions

Abundant responsive regulatory elements were found in the promoter regions of *CsPP2Cs* through PlantCARE analysis (Fig. 5). The cis-elements screened were divided into two categories. The first type of element was hormone response, such as TCA-element (salicylic acid response element), the ABRE (ABA response element), the TGA-element (auxin response element), the CGTCA-motif and TGACG-motif (MeJA-responsiveness response element), and the P-box (gibberellin response element), among others. The second type of element was stress response, such as LTR, MYC, TC-rich repeats and MBS. The abscisic acid ABA-responsive (ABRE) elements were identified abundantly in the promoter regions of *CsPP2Cs*, among which *CsPP2C2* and *CsPP2C3* contained 12 ABREs, which was the largest number, followed by *CsPP2C7*, *CsPP2C20*, *CsPP2C26*, *CsPP2C40*, and *CsPP2C56*; it was the most abundant element in the promoter region. The second was the MYC element, which was not present in only *CsPP2C12*–*13, CsPP2C32*–*34*, *CsPP2C42*–*43*, *CsPP2C45*, *46*, and *48*, and was most abundant in *CsPP2C1*. This suggests that most *CsPP2C* genes may respond to various abiotic stresses.

### Tissue-specific expression profiles of *CsPP2C* genes

**Fig. 6:**
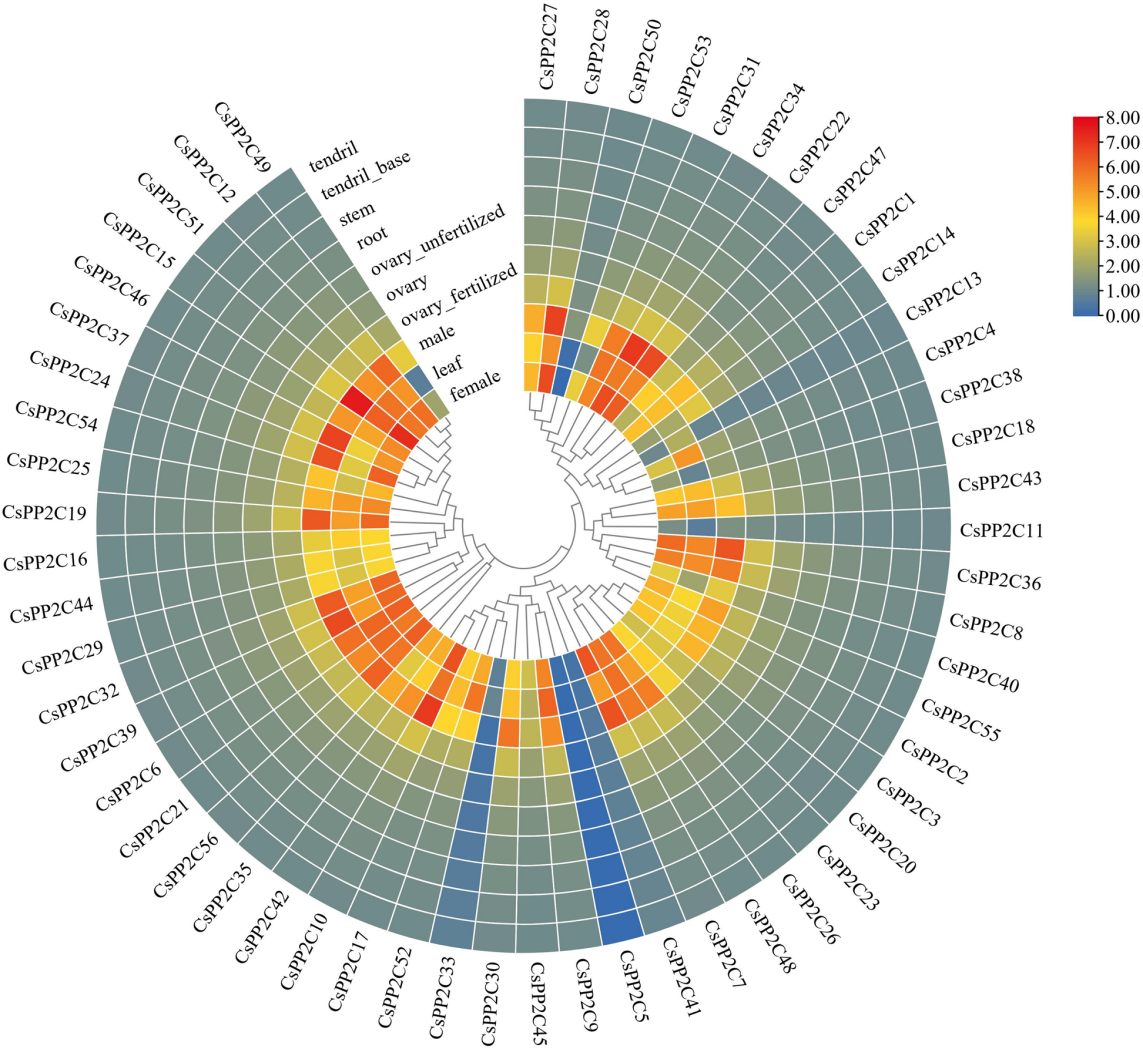
Expression patterns of *CsPP2C* genes in various wheat tissues. Heatmap of *CsPP2C* RNA-seq data in five tissues at three different developmental stages was created by TBTools. The expression values mapped to a color gradient from low (blue) to high expression (orange) are shown at the right of the figure

To better understand the role of *CsPP2C* genes in cucumber growth and development, the temporal and spatial expression patterns of *CsPP2C* genes was analyzed using RNA-Seq data of different tissues of the cucumber cultivar Chinese long ‘9930’ (Fig. 6). Only *CsPP2C11*, *41*, *5*, *33,* and *50* had low expression in all 10 tissues. On the contrary, the expression levels of other *CsPP2C* genes were high in the fertilized ovaries, male, female, and leaf but low in other organs, such as *CsPP2C12*, *51*, *15*, *46*, *37*, *31*, *22*, and *47.* Moreover, the expression level of *CsPP2C49* was medium in males but low in other tissues. Similarly, the expression level of *CsPP2C53* was medium in females and males but low in other tissues. In general, most cucumber *CsPP2C* genes showed similar expression patterns in different tissues.

### Changes in relative expression of the *CsPP2C* gene under osmotic stress and ABA treatment

**Fig. 7:**
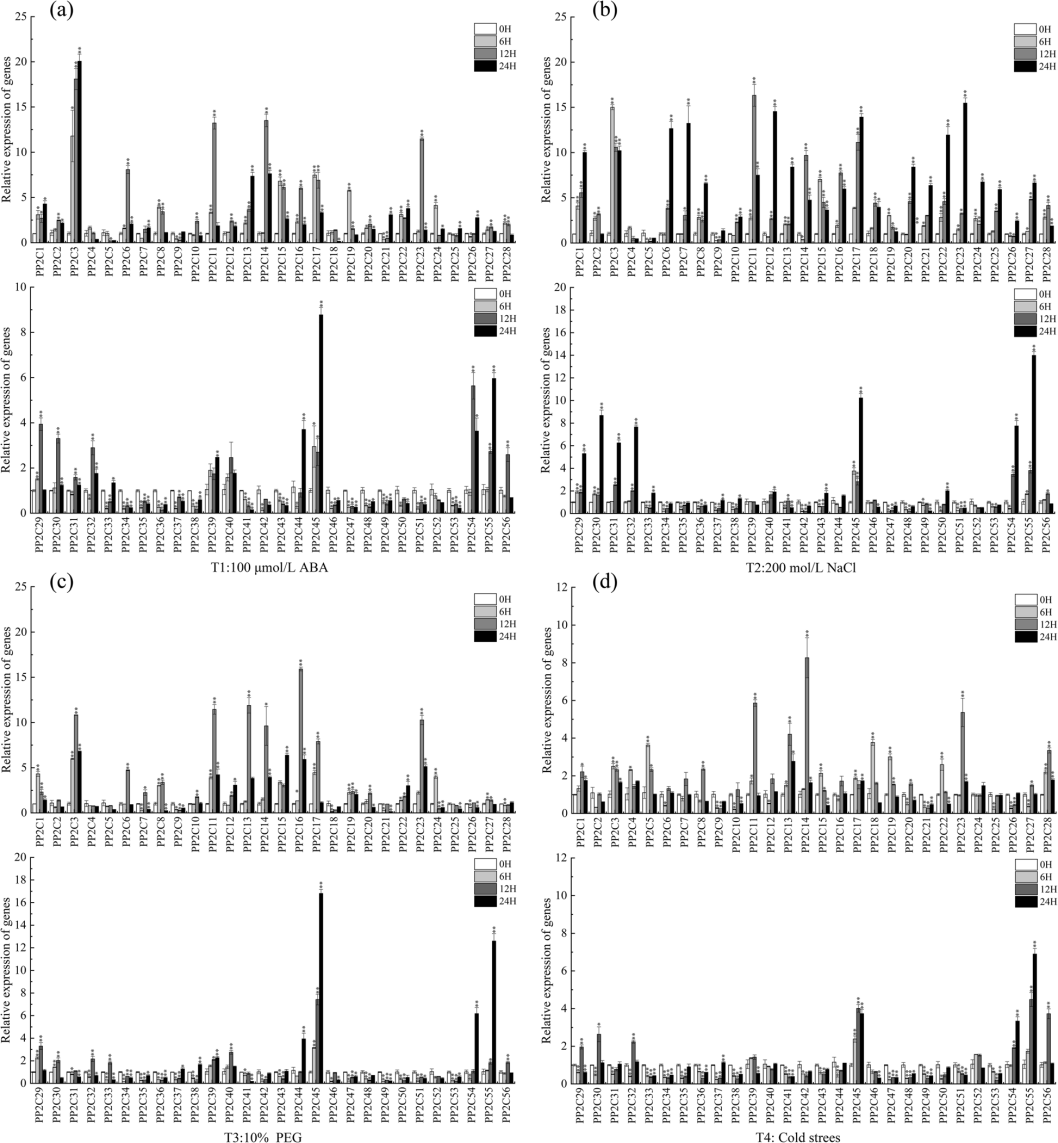
Quantitative real-time PCR analysis of *CsPP2C* genes in response to ABA (**a**), NaCl (**b**), PEG (**c**), cold treatments (**d**). he gene relative expression was calculated using the 2^-∆∆Ct^ method with CsActin as an internal control, and value represents mean ± SE of three biological replicates. Statistical analyses were carried out by student’s *t*-test to determine the differences in gene abundances between 0 h and other treatment times. Asterisks indicated values that are significantly different from CK (0 h) (* *p* < 0.05, ** *p* < 0.01, one-way ANOVA)

Several members of group A *PP2Cs* have been shown to function as negative regulators of the ABA signaling pathway in *Arabidopsis.* The expression of seven *PP2C* genes in *Arabidopsis* was suppressed by ABA treatment, two of which are members of subfamily D. The *PP2C* genes in different subfamilies might play different functional roles in distinct signaling pathways. Therefore, the expression of 56 *CsPP2C* genes under ABA, salt, drought, and cold treatment was analyzed by qRT-PCR. Under ABA treatment (Fig. 7 a), we observed that the expression of *CsPP2C1*–*3*, *13*, *22*, 2*9*, *39*, *45*, *54*, and *55* continually increased with the extension of ABA treatment time. Among them, the expression level of *CsPP2C3* was the highest at 24 h, 20 times that at 0 h. On the contrary, the expression levels of *CsPP2C4*, *5*, 9, *33*–*38*, *41*–*43*, and *46*–*53* had decreasing trends. The expression levels of *CsPP2C6*, *8*, *11*, *14*–*17*, *19*, *20*, *23*, 27–*29*, and *40* were increased at 6 and 12 h but decreased at 24 h; among them, *CsPP2C14*–*17* was up-regulated most significantly, and although their expression decreased at 24 h, was still higher than that at 0 h. Compared with 0 h, the expression of *CsPP2C19*, *24* was up-regulated at 6 h, the expression of *CsPP2C10*, *12*, *24*, *30*, and *32* was up-regulated at 12 h, while the expression of *CsPP2C25* and *31* was almost unchanged. As shown in Fig. 7 b, under 10% PEG treatment, *CsPP2C1*, *3*, *11*–*17, 23*, *45*, and *55* were significantly up-regulated, among which *CsPP2C3*, *CsPP2C11*–*17* and *CsPP2C45* were up-regulated most noticeably and were more than eight times higher than that at 0 h after 6 h treatment, especially *CsPP2C45*, which was more than 16 times higher than that at 0 h after 24 h treatment. The relative expression levels of other genes such as *CsPP2C9*, *18*, *21*, *34*–*36,* and *46*–*53* were down-regulated. It is worth noting that after 200 mmol/L NaCl treatment (Fig. 7 c), the expression of many genes increased significantly after 12 and 24 h treatment and were higher than other treatments, especially *CsPP2C1*, *CsPP2C6*–*8*, *CsPP2C11*, *CsPP2C12*, *CsPP2C17*, and *CsPP2C20*–*32*. The highest was more than 15 times higher than that at 0 h after 24 h treatment (*CsPP2C23*). The relative expression of other genes was similar to that of drought treatment. Interestingly, the expression of almost all *CsPP2C* genes under cold stress (Fig. 7 d) was lower than that of other treatments, except for the genes whose expression was down-regulated, such as *CsPP2C1*, *3*, *11–17*, *23*, *45*, and *54*–*55*. Compared with that at 0 h, the up-regulation of these genes was not high, and *CsPP2C14* had the highest up-regulation range at 12 h, more than eight times that of 0 h. Under ABA, drought, and salt treatments, the expression level of *CsPP2C3*, *11–17*, *23*, *45*, *54*, and *55* was significantly up-regulated. This may play an important role in abiotic stress. These results provide a basis for the functional study of the *PP2C* gene in the future.

## Discussion

Plant PP2Cs play a role in plant development as well as in drought, salt, alkali, and fungal pathogen stress resistance [24, 35] and are important PKs in the ABA signaling pathway [36]. In recent years, they have been identified in several plants, such as *Arabidopsis* [16], rice [16], maize [29], and wheat [37]. In this study, we comprehensively analyzed *CsPP2C* genes in cucumber, including genome-wide identification, chromosome location, collinear relationship, gene structure, conserved motifs, and expression patterns. Fifty-six cucumber *PP2C* genes were identified by homology comparison. Compared with *Arabidopsis* (80) [16], wheat (95) [37], maize (97) [16, 38], rice (78) [16] and *B. distachyon* (86) [12], the amount of *PP2C* in cucumber was much less. This indicated that the increase and expansion of *PP2C* genes are diverse in different species and may also be related to their adaptation to complex environments or to the few chromosomes and small genome of cucumber (2n = 2x = 14) [39].

Chromosome mapping (Fig. 1) found that the *PP2C* genes located on Chr 3, 4, and 6 were the most distributed, with the number of 13, 9 and 11, respectively. The *CsPP2C* gene does not cluster on the chromosome, and we did not find tandem duplicate gene pairs. Collinearity results (Fig. 2 a) showed that seven homologous gene pairs existed among *CsPP2Cs*, and there were 59 pairs of collinear genes in *Arabidopsis* and cucumber (Fig. 2 b), indicating that *CsPP2C* genes expand primarily through segmental duplication of the chromosome. Compared with tandem repeats, segmental duplication is more conducive to the maintenance of gene function in the process of gene replication [40]. In addition, the amplification of *AtPP2C* genes in *Arabidopsis* is also through fragment replication [41], which was consistent with our results. Furthermore, the d_N_/d_S_ values of these gene replication pairs were all less than 1, indicating that they underwent purification selection, and their high conservatism in the evolution process was predicted (Table S1).

According to the phylogenetic tree, the *PP2C* genes of cucumber and *Arabidopsis* were divided into 13 groups (Fig. 3). This grouping is the same as that in *Arabidopsis*, rice, and wheat [16, 37]. Phylogenetic tree analysis showed that most subfamilies contained both cucumber and *Arabidopsis* genes. Cucumber and *Arabidopsis* had a similar number of members in the same subfamily, and the members of the two species tended to cluster separately. In addition, we analyzed the gene structure and conserved motifs of *CsPP2C* genes. The exon/intron structure of genes is an important marker of the evolutionary relationship among gene family members [42]. Our results indicated that cucumber *PP2C* genes in the same group had similar exon/intron structures (Fig. 4 b). While the distribution pattern of exon/intron structures was similar to most genes in the same group, there were some exceptions, which may be due to a variety of reasons [43]. Unlike other genes, *CsPP2C14*, *40*, *3*, *24*, *37*, *15* and *51* have no non-coding region. In addition, all *CsPP2C* genes contain different numbers of exons and introns, of which *CsPP2C35* has the longest coding sequence and *CsPP2C21* has the largest number of exons. A total of 10 conserved motifs were identified in the amino acid sequence of *CsPP2C* genes. The results (Fig. 4 c) showed that *CsPP2C* genes in the same group showed similar motif distribution, and motifs 1, 2 (except *CsPP2C3*, *23*), 3 (except *CsPP2C51*), 4, 6 and 7 existed in all cucumber *PP2C* genes. This motif pattern was closely related to the catalytic core domain of the PP2C protein [44].

Understanding the subcellular location of proteins (Table 1) may provide us with necessary information to infer the biological function of proteins. *CsPP2Cs* were primarily located in the nucleus, cytoplasm and chloroplast, thus it is speculated that they are related to photosynthesis, respiration, and cell growth and development. At the same time, the *CsPP2C* gene also showed specific expression in different tissues (Fig. 6). The expression of all *CsPP2C* genes in female, leaf, male, and fertilized ovaries was higher (except *CsPP2C5*, *33*, and *41*), but lower in other tissues. In cotton, the majority of the *GhPP2CAs* was predominantly expressed in flowers [45]. In *Arabidopsis* and rice, most *AtPP2C* genes (84%) and *OsPP2C* genes (72%) are expressed in more than two tissues [16]. In maize, most *ZmPP2Cs* had a very broad expression spectrum; only *ZmPP2C51*, *ZmPP2C87*, *ZmPP2C86*, *ZmPP2C80*, *ZmPP2C58* and *ZmPP2C41* were low or not expressed in different tissues [29]. This shows that most *PP2C* gene family members in different plants play a role in multiple processes of plant growth and development, and that only a small number of members act in tissue-specific biological processes.

Cis-acting elements are important regulators of hormone response in plant development and resistance to various stresses [46]. In this study, ABRE elements were most abundant in all *CsPP2C* gene promoter regions, followed by MYC elements (Fig. 5). qRT-PCR results (Fig. 7) showed that *CsPP2C1*, *3*, *6*, *8*, *11–17*, *22*, *23*, *45*, *54*, and *55* responded significantly to ABA and drought treatment; *CsPP2C1*, *3*, *6*, *8*, *11*, *14*–*17*, *54* and *55* contain many MYC elements; and *CsPP2C3*, 13, *15*–*17*, *22*, and *23* contain many ABA response elements. These results indicated that the number of homeopathic elements in the promoter region of *CsPP2C* genes may be related to its response to stress.

Studies on *Arabidopsis* and rice suggest that family A *PP2C* plays an important role in plant responses to abiotic stress, especially in ABA signaling [47]. Overexpression of group E *PP2C* gene, *AtPP2CF1*, increased plant biomass in *Arabidopsis* [48]. *AtPP2CG1* in group G is an ABA-dependent positive regulator of salt tolerance [49]. In addition, the expression patterns of *PP2C* genes in maize [29], rice [16] and *B. distachyon* [12] under various stresses have been examined. In *B.distachyon*, *BdPP2C70* from subgroup D, *BdPP2C13* from subgroup F and *BdPP2C32* from subgroup G exhibited strongly increased expression levels in response to ABA and abiotic treatments [12]. In rice, the expression levels of all *OsPP2C* genes in subgroup A increased after ABA and salt treatment [16]. Our study showed that (Fig. 7) the relative expressions of *CsPP2C3* from subgroup A, *CsPP2C14*, *CsPP2C13*, from subgroup B, *CsPP2C12*, *CsPP2C15*, *CsPP2C54* from subgroup D, *CsPP2C17*, *CsPP2C45* from subgroup E, *CsPP2C55* from subgroup G, and *CsPP2C23* from subgroup I were up-regulated under the four treatments. In addition, the expression of many *CsPP2C* genes showed a similar trend under four different treatments, which was consistent with research results for *Medicago truncatula* [50]. In cucumber, subgroup A *CsPP2Cs* includes seven members (*CsPP2C2*, *CsPP2C3*, *CsPP2C7*, *CsPP2C20*, C*s*PP2C26, *CsPP2C41*, and *CsPP2C48*). The qRT-PCR results suggested that only *CsPP2C3* was highly induced by exogenous ABA treatment, and that *CsPP2C41* and *CsPP2C48* showed a downward trend, while other genes were up-regulated but not noticeable relative to 0 h. In eight members of *B.distachyon* subgroup A, *BdPP2C27* and *BdPP2C34* were also insensitive to ABA treatment. These results showed that not all *CsPP2C* genes homologous to group A members of *Arabidopsis* responded to ABA treatment as well as *CsPP2C* genes in other groups, and that there may be different expression patterns of *PP2C* genes in different species. Under cold stress, the relative expression level of most *CsPP2C* genes was lower (Fig. 7d), which was not significant, compared with that of other treatments. Many genes are silenced or reduced in expression. This indicated that the *PP2C* gene of cucumber had a weak response to cold stress. In conclusion, *CsPP2C* genes in different groups have different responses to different stresses, and there were *CsPP2CA*s genes that did not respond to ABA treatment. The expression of *CsPP2C3*, *11–17*, *23*, *45*, *54*, and *55* was significantly up-regulated under the four treatments, suggesting that these genes from different subgroups may play an important role in cucumber resistance to abiotic stress. These results provide a reference for the study of *PP2C* under different stresses; their functions need to be further explored.

## Conclusion

In this study, the whole genome of the cucumber *CsPP2C* gene family was identified, and its expression level was analyzed. Fifty-six *CsPP2C* genes were highly similar in gene structure and had conserved motifs. Collinearity and selection pressure indicated that *CsPP2C* genes were amplified by fragment replication and underwent purifying selection during evolution, ensuring the stability of their functions. In addition, qRT-PCR results showed that in subgroup A, only *CsPP2C3* responded significantly to ABA and other treatments, other genes were also up-regulated but not significant, while the expressions of *CsPP2C41* and *CsPP2C48* were down-regulated. In addition, the members of other subgroups also have genes that respond significantly to abiotic stress, such as *CsPP2C11–17*, *CsPP2C23*, *45*, *54*, and *55.* These genes may play an important role in cucumber growth and development. This study provided relevant information for follow-up study of *PP2C* gene function in cucumber.

## Methods

### Plant materials and growth conditions

The germinated seeds (*Cucumis sativus* L, “L306” cultivar) were treated by 5% NaClO for 10 min and washed with deionized water 3–5 times. Afterwards, the cucumber seeds were put into an artificial climate incubator to promote germination. The cultivation conditions were as follows: relative humidity 80%, temperature 25 °C/18 °C (day/night), with a light intensity during the day of 250 μmol m^− 2^ s^− 1^. After 4 days of culture, cucumber seedlings were soaked in Yamazaki nutrient solution for hydroponics. When cucumber seedlings grew to three true leaves, four treatments were set, T1: 100 μmol/L ABA; T2: 10% PEG; T3: 200 mmol/L NaCl; T4: cold stress. Cucumber leaves after 0, 6, 12 and 24 h of different treatments were collected, and the samples were put into liquid nitrogen and immediately stored in a refrigerator at − 80 °C.

### Identification of *PP2C* genes in cucumber

The protein sequences of 80 *PP2C* genes in *Arabidopsis* were downloaded from the *Arabidopsis* information resource website (https://www.arabidopsis.org/). Then, cucumber genome data (http://cucurbitgenomics.org/) was used for BLASTp. Next, screening of candidate genes was performed, and the retrieval threshold was set as E-value < E^− 10^. Redundant results were removed manually. At the same time, the protein sequences of the *Arabidopsis PP2C* gene were compared in TBtools to further screen candidate genes. Then, the Pfam (http://pfam.xfam.org/search#tabview=tab1) and NCBI-CDD databases (https://www.ncbi.nlm.nih.gov/cdd/) were used for domain identification of candidate gene sequence structures. Candidate genes that did not contain the specific domain of the *PP2C* gene (registration number: PF00481) were manually removed [51].

### Sequence analysis and basic information of the cucumber *PP2C* gene family

The subcellular localization of the PP2C protein in cucumber was predicted by Wolf PSORT (https://wolfpsort.hgc.jp/) [52]. The number of amino acids and isoelectric genes were identified using ExPASy (websitehttps://web.expasy.org/protparam/) [53]. Additionally, the secondary structure of the cucumber PP2C protein were predicted through the online web site (https://npsa-prabi.ibcp.fr/cgi-bin/npsa_automat.pl? page = npsa_sopma. html).

### Analysis of chromosome location and collinearity analysis

The Chinese long ‘9930’ v3 gff3 file was downloaded from the cucumber genome database, and the chromosome location and length information of 56 *PP2C* genes were screened for and classified as *CsPP2Cs* according to their distribution on chromosomes. MG2C (http://mg2c.iask.in/mg2c_v2.0/) was used to map the chromosomal location. Using MCScanX search for homology, the protein-coding genes from the cucumber genome were compared against themselves and those from *Arabidopsis* genomes using BLASTp, and the retrieval threshold was set as E-value < E^− 5^. Others were not modified by default parameters. Whole-genome BLASTp results were used to compute collinear blocks for all possible pairs of chromosomes and scaffolds [54]. Subsequently, TBtools was used to highlight the identified *PYL* collinear pairs and their collinear pairs with *Arabidopsis* [55].

### Selective pressure analysis of the cucumber *PP2C* genes family

PAL2NAL (*http://www.bork.embl.de/pal2nal/index.cgi?*) was used to calculate the non-synonymous/synonymous (d_N_/d_s_) value of duplicate gene pairs [56].

### Construction of phylogenetic tree

The phylogenetic tree of the *PP2C* gene family of cucumber and *Arabidopsis thaliana* was constructed using MEGA 7 with muscle-sequence alignment methods selected and the Bootstrap value set as 1000 [57]. Finally, a stable minimum neighbor tree was selected to represent their evolutionary relationship and the constructed tree was beautified using iTOL (https://itol.embl.de/).

### Analysis of gene exon-intron structures and protein conserved motifs

The structure of the *PP2C* gene in cucumber was analyzed by GSDS (http://gsds.gao-lab.org/) [58]. The conserved motif of the cucumber *PP2C* gene was analyzed by MEME (http://meme-suite.org/tools/meme) [59]. The maximum motif number was set as 10, and the remaining parameters were set as default values.

### Analysis of cis-acting elements in *CsPP2C* gene promoters

The first 2000 bp upstream sequence of 56 identified *PP2C* gene initiation codons (ATG) was extracted using TBtools. The 2000 bp sequence was then submitted to PlantCARE (http://bioinformatics.psb.ugent.be/ webtools/plantcare/html/) for cis role element forecast analysis [60].

### Tissue expression analysis of *PP2C* genes

To study the gene-specific expression of *CsPP2C* genes in different tissues of cucumber, the accession number PRJNA80169 was used from cucumber genome data (http://cucurbitgenomics.org/) to obtain cucumber RNA samples from different tissue and organ (tendril-base, tendril, root, leaf, stems, ovary-unfertilized, ovary-fertilized, ovary) RNA-Seq data [61]. Finally, the log2 method was used for data conversion and TBtools was used to draw the expression heat map of the *CsPP2*C genes.

### RNA extraction and real-time PCR (qRT-PCR)

Total RNA was isolated from collected samples using a Plant RNA Extraction kit (Tiangen, China). The complementary DNA was synthesized using the fastking cDNA dispersing RT supermaxs kit (Tiangen, China) with 2 μL RNA as the template. The coding sequences of *CsPP2C* genes were input into the homepage of Shanghai biology company (Shanghai, China) for online primer design (Table S2). The SYBR Green kit (Tiangen, China) was used for fluorescence quantitative analysis. The volume of the reaction system was 20 μL, containing 2 μL cDNA solution, 10 μL 2*SuperReal PreMix Plus, 0.6 μL of 10 μM forward and reverse primers, 0.4 μL 50*ROX Reference Dye, and 6.4 μL of distilled deionized water. Next, qRT-PCR was performed using the LightCycler® 480 II real-time fluorescence quantitative PCR instrument. The amplification program conditions were as follows: 95 °C for 15 min, 40 cycles of 95 °C for 10 s and 60 °C for 30 s. Each sample was replicated three times. The relative expressions levels of the *PP2C* genes were calculated using the 2^-∆∆CT^ method [62]. SPSS 20.0 was used to analyze the relative expressions and Origin 9.0 was used to complete the histogram of relative expressions.

## Supplementary Information

**Additional file 1: Table S1.** Selective pressure analysis of *PP2C* genes family.

**Additional file 2: Table S2.** Conserved motifs in the amino acid sequences of *CsPP2C* proteins.

**Additional file 3: **Mean value, SE and T-test of relative expression of PP2C genes.

## Data Availability

The qRT-PCR data supporting the gene relative expression results of this study can be found in supplementary material 1. The login numbers of all CsPP2C genes identified in the experiment can be obtained in the cucumber (Chinese Long) v3 genome database (http://cucurbitgenomics.org/organism/20).

## Declarations

All methods were carried out in accordance with relevant guidelines and regulations.

## References

[1] Wang L, Liang S, Lu Y-T (2001). Characterization, physical location and expression of the genes encoding calcium/calmodulin-dependent protein kinases in maize (*Zea mays* L.). Planta.

[2] Kong X, Lv W, Jiang S, Zhang D, Cai G, Pan J, Li D (2013). Genome-wide identification and expression analysis of calcium-dependent protein kinase in maize. BMC Genomics.

[3] Li F, Fan G, Wang K, Sun F, Yuan Y, Song G, Li Q, Ma Z, Lu C, Zou C (2014). Genome sequence of the cultivated cotton Gossypium arboreum. Nat Genet.

[4] Cohen P (1989). The structure and regulation of protein phosphatases. Annu Rev Biochem.

[5] Luan S (2003). Protein phosphatases in plants. Annu Rev Plant Biol.

[6] MacKintosh C, Coggins J, Cohen P (1991). Plant protein phosphatases. Subcellular distribution, detection of protein phosphatase 2C and identification of protein phosphatase 2A as the major quinate dehydrogenase phosphatase. Biochem J.

[7] Zhou H-W, Nussbaumer C, Chao Y, DeLong A (2004). Disparate roles for the regulatory a subunit isoforms in Arabidopsis protein phosphatase 2A. Plant Cell.

[8] Smith RD, Walker JC (1993). Expression of multiple type 1 phosphoprotein phosphatases in Arabidopsis thaliana. Plant Mol Biol.

[9] Takemiya A, Kinoshita T, Asanuma M, Shimazaki K-I (2006). Protein phosphatase 1 positively regulates stomatal opening in response to blue light in Vicia faba. Proc Natl Acad Sci.

[10] Lin Q, Li J, Smith RD, Walker JC (1998). Molecular cloning and chromosomal mapping of type one serine/threonine protein phosphatases in *Arabidopsis thaliana*. Plant Mol Biol.

[11] Zimmerlin A, Jupe SC, Bolwell GP (1995). Molecular cloning of the cDNA encoding a stress-inducible protein phosphatase 1 (PP1) catalytic subunit from French bean (*Phaseolus vulgaris* L.). Plant Mol Biol.

[12] Cao J, Jiang M, Li P, Chu Z (2016). Genome-wide identification and evolutionary analyses of the PP2C gene family with their expression profiling in response to multiple stresses in *Brachypodium distachyon*. BMC Genomics.

[13] Song S-K, Hofhuis H, Lee MM, Clark SE (2008). Key divisions in the early *Arabidopsis* embryo require POL and PLL1 phosphatases to establish the root stem cell organizer and vascular axis. Dev Cell.

[14] Schweighofer A, Hirt H, Meskiene I (2004). Plant *PP2C* phosphatases: emerging functions in stress signaling. Trends Plant Sci.

[15] Meskiene I, Baudouin E, Schweighofer A, Liwosz A, Jonak C, Rodriguez PL, Jelinek H, Hirt H (2003). Stress-induced protein phosphatase 2C is a negative regulator of a mitogen-activated protein kinase. J Biol Chem.

[16] Xue T, Wang D, Zhang S, Ehlting J, Ni F, Jakab S, Zheng C, Zhong Y (2008). Genome-wide and expression analysis of protein phosphatase 2C in rice and *Arabidopsis*. BMC Genomics.

[17] Schweighofer A, Kazanaviciute V, Scheikl E, Teige M, Doczi R, Hirt H, Schwanninger M, Kant M, Schuurink R, Mauch F (2007). The PP2C-type phosphatase *AP2C1*, which negatively regulates *MPK4* and *MPK6*, modulates innate immunity, jasmonic acid, and ethylene levels in *Arabidopsis*. Plant Cell.

[18] Gagne JM, Clark SE (2010). The *Arabidopsis* stem cell factor POLTERGEIST is membrane localized and phospholipid stimulated. Plant Cell.

[19] Chan Z (2012). Expression profiling of ABA pathway transcripts indicates crosstalk between abiotic and biotic stress responses in *Arabidopsis*. Genomics.

[20] Merlot S, Gosti F, Guerrier D, Vavasseur A, Giraudat J (2001). The ABI1 and ABI2 protein phosphatases 2C act in a negative feedback regulatory loop of the abscisic acid signalling pathway. Plant J.

[21] Boudsocq M, Barbier-Brygoo H, Lauriere C (2004). Identification of nine sucrose nonfermenting 1-related protein kinases 2 activated by hyperosmotic and saline stresses in *Arabidopsis thaliana*. J Biol Chem.

[22] Smékalová V, Doskočilová A, Komis G, Šamaj J (2014). Crosstalk between secondary messengers, hormones and MAPK modules during abiotic stress signalling in plants. Biotechnol Adv.

[23] Wu P, Wang W, Li Y, Hou X (2017). Divergent evolutionary patterns of the MAPK cascade genes in Brassica rapa and plant phylogenetics. Hortic Res.

[24] Chen C, Yu Y, Ding X, Liu B, Duanmu H, Zhu D, Sun X, Cao L, Li Q, Zhu Y (2018). Genome-wide analysis and expression profiling of *PP2C* clade D under saline and alkali stresses in wild soybean and *Arabidopsis*. Protoplasma.

[25] Haider MS, Kurjogi MM, Khalil-Ur-Rehman M, Fiaz M, Pervaiz T, Jiu S, Haifeng J, Chen W, Fang J (2017). Grapevine immune signaling network in response to drought stress as revealed by transcriptomic analysis. Plant Physiol Biochem.

[26] Galbiati M, Simoni L, Pavesi G, Cominelli E, Francia P, Vavasseur A, Nelson T, Bevan M, Tonelli C (2008). Gene trap lines identify Arabidopsis genes expressed in stomatal guard cells. Plant J.

[27] Lee MW, Jelenska J, Greenberg JT (2008). *Arabidopsis* proteins important for modulating defense responses to pseudomonas syringae that secrete HopW1-1. Plant J.

[28] Singh A, Giri J, Kapoor S, Tyagi AK, Pandey GK (2010). Protein phosphatase complement in rice: genome-wide identification and transcriptional analysis under abiotic stress conditions and reproductive development. BMC Genomics.

[29] Fan K, Yuan S, Chen J, Chen Y, Li Z, Lin W, Zhang Y, Liu J, Lin W (2019). Molecular evolution and lineage-specific expansion of the *PP2C* family in *Zea mays*. Planta.

[30] Singh A, Jha SK, Bagri J, Pandey GK (2015). ABA inducible rice protein phosphatase 2C confers ABA insensitivity and abiotic stress tolerance in *Arabidopsis*. PLoS One.

[31] Liu L, Hu X, Song J, Zong X, Li D, Li D (2009). Over-expression of a *Zea mays* L. protein phosphatase 2C gene (*ZmPP2C*) in *Arabidopsis thaliana* decreases tolerance to salt and drought. J Plant Physiol.

[32] Jia H-f, Lu D, Sun J-H, Li C-I, Xing Y, Qin L, Shen Y-Y (2013). Type 2C protein phosphatase ABI1 is a negative regulator of strawberry fruit ripening. J Exp Bot.

[33] Cheung J, Estivill X, Khaja R, MacDonald JR, Lau K, Tsui L-C, Scherer SW (2003). Genome-wide detection of segmental duplications and potential assembly errors in the human genome sequence. Genome Biol.

[34] Yadav CB, Bonthala VS, Muthamilarasan M, Pandey G, Khan Y, Prasad M (2015). Genome-wide development of transposable elements-based markers in foxtail millet and construction of an integrated database. DNA Res.

[35] Akimoto-Tomiyama C, Tanabe S, Kajiwara H, Minami E, Ochiai H (2018). Loss of chloroplast-localized protein phosphatase 2Cs in *Arabidopsis thaliana* leads to enhancement of plant immunity and resistance to Xanthomonas campestris pv. Campestris infection. Mol Plant Pathol.

[36] Park S-Y, Fung P, Nishimura N, Jensen DR, Fujii H, Zhao Y, Lumba S, Santiago J, Rodrigues A, Tsz-fung FC (2009). Abscisic acid inhibits type 2C protein phosphatases via the PYR/PYL family of START proteins. Science.

[37] Yu X, Han J, Wang E, Xiao J, Hu R, Yang G, He G (2019). Genome-wide identification and homoeologous expression analysis of PP2C genes in wheat (*Triticum aestivum* L.). Front Genet.

[38] Wei K, Pan S (2014). Maize protein phosphatase gene family: identification and molecular characterization. BMC Genomics.

[39] Yang L, Koo DH, Li Y, Zhang X, Luan F, Havey MJ, Jiang J, Weng Y (2012). Chromosome rearrangements during domestication of cucumber as revealed by high-density genetic mapping and draft genome assembly. Plant J.

[40] Lynch M, Conery JS (2000). The evolutionary fate and consequences of duplicate genes. Science.

[41] Cannon SB, Mitra A, Baumgarten A, Young ND, May G (2004). The roles of segmental and tandem gene duplication in the evolution of large gene families in *Arabidopsis thaliana*. BMC Plant Biol.

[42] Long M, Betrán E, Thornton K, Wang W (2003). The origin of new genes: glimpses from the young and old. Nat Rev Genet.

[43] Rogozin IB, Sverdlov AV, Babenko VN, Koonin EV (2005). Analysis of evolution of exon-intron structure of eukaryotic genes. Brief Bioinform.

[44] Shi Y (2009). Serine/threonine phosphatases: mechanism through structure. Cell.

[45] Lu T, Zhang G, Wang Y, He S, Sun L, Hao F (2019). Genome-wide characterization and expression analysis of *PP2CA* family members in response to ABA and osmotic stress in *Gossypium*. PeerJ.

[46] Nishimura N, Sarkeshik A, Nito K, Park SY, Wang A, Carvalho PC, Lee S, Caddell DF, Cutler SR, Chory J (2010). PYR/PYL/RCAR family members are major in-vivo ABI1 protein phosphatase 2C-interacting proteins in *Arabidopsis*. Plant J.

[47] Bhaskara GB, Nguyen TT, Verslues PE (2012). Unique drought resistance functions of the highly ABA-induced clade a protein phosphatase 2Cs. Plant Physiol.

[48] Sugimoto H, Kondo S, Tanaka T, Imamura C, Muramoto N, Hattori E, Ki O, Mitsukawa N, Ohto C (2014). Overexpression of a novel Arabidopsis PP2C isoform, AtPP2CF1, enhances plant biomass production by increasing inflorescence stem growth. J Exp Bot.

[49] Liu X, Zhu Y, Zhai H, Cai H, Ji W, Luo X, Li J, Bai X (2012). *AtPP2CG1*, a protein phosphatase 2C, positively regulates salt tolerance of Arabidopsis in abscisic acid-dependent manner. Biochem Biophys Res Commun.

[50] Yang Q, Liu K, Niu X, Wang Q, Wan Y, Yang F, Li G, Wang Y, Wang R (2018). Genome-wide identification of PP2C genes and their expression profiling in response to drought and cold stresses in *Medicago truncatula*. Sci Rep.

[51] Finn RD, Clements J, Eddy SR (2011). HMMER web server: interactive sequence similarity searching. Nucleic Acids Res.

[52] Xiong E, Zheng C, Wu X, Wang W (2016). Protein subcellular location: the gap between prediction and experimentation. Plant Mol Biol Report.

[53] Artimo P, Jonnalagedda M, Arnold K, Baratin D, Csardi G, De Castro E, Duvaud S, Flegel V, Fortier A, Gasteiger E (2012). ExPASy: SIB bioinformatics resource portal. Nucleic Acids Res.

[54] Wang Y, Tang H, DeBarry JD, Tan X, Li J, Wang X, Lee T-H, Jin H, Marler B, Guo H (2012). MCScanX: a toolkit for detection and evolutionary analysis of gene synteny and collinearity. Nucleic Acids Res.

[55] Chen C, Chen H, Zhang Y, Thomas HR, Frank MH, He Y, Xia R (2020). TBtools: an integrative toolkit developed for interactive analyses of big biological data. Mol Plant.

[56] Goldman N, Yang Z (1994). A codon-based model of nucleotide substitution for protein-coding DNA sequences. Mol Biol Evol.

[57] Kumar S, Stecher G, Tamura K (2016). MEGA7: molecular evolutionary genetics analysis version 7.0 for bigger datasets. Mol Biol Evol.

[58] Hu B, Jin J, Guo A-Y, Zhang H, Luo J, Gao G (2015). GSDS 2.0: an upgraded gene feature visualization server. Bioinformatics.

[59] Bailey TL, Boden M, Buske FA, Frith M, Grant CE, Clementi L, Ren J, Li WW, Noble WS (2009). MEME SUITE: tools for motif discovery and searching. Nucleic Acids Res.

[60] Lescot M, Déhais P, Thijs G, Marchal K, Moreau Y, Van de Peer Y, Rouzé P, Rombauts S (2002). PlantCARE, a database of plant cis-acting regulatory elements and a portal to tools for in silico analysis of promoter sequences. Nucleic Acids Res.

[61] Li Z, Zhang Z, Yan P, Huang S, Fei Z, Lin K (2011). RNA-Seq improves annotation of protein-coding genes in the cucumber genome. BMC Genomics.

[62] Livak KJ, Schmittgen TD (2001). Analysis of relative gene expression data using real-time quantitative PCR and the 2− ΔΔCT method. Methods.

