# Supplementary material for: Genome-wide identification and expression analysis of the cucumber PP2C gene family
Source: BMC Genomics. 2022 Aug 6;23:563. doi: 10.1186/s12864-022-08734-y (PMC9356470; doi:10.1186/s12864-022-08734-y)
Supplement: Supplementary file 1 — Additional file 1: Table S1. Selective pressure analysis of PP2C genes family. [file 12864_2022_8734_MOESM1_ESM.doc]

**Table S1** Selective pressure analysis of *PP2C* genes family.

| **species** | **A pair of genes** | **S** | **N** | **ds** | **d_N_** | **d_N_/d_s_** |
| --- | --- | --- | --- | --- | --- | --- |
| **cucumber** | *CsPP2C5*-*CsPP2C9* | 286.3 | 793.7 | 1.2987 | 0.2539 | 0.1955 |
|  | *CsPP2C3*-*CsPP2C41* | 233.7 | 699.3 | 50.0625 | 0.6171 | 0.0123 |
|  | *CsPP2C12*-*CsPP2C49* | 287.7 | 903.3 | 1.7940 | 0.1444 | 0.0805 |
|  | *CsPP2C24*-*CsPP2C37* | 276.4 | 827.6 | 2.1948 | 0.1881 | 0.0857 |
|  | *CsPP2C18*-*CsPP2C43* | 232.4 | 928.6 | 2.0387 | 0.2006 | 0.0984 |
|  | *CsPP2C15*-*CsPP2C49* | 293.4 | 867.6 | 1.4963 | 0.1448 | 0.0968 |
|  | *CsPP2C29*-*CsPP2C44* | 494.6 | 1575.4 | 3.0854 | 0.3221 | 0.1044 |
| **cucumber and *Arabidopsis*** | *AtPP2C8*-*CsPP2C1* | 249.3 | 770.7 | 4.6543 | 0.2222 | 0.0477 |
|  | *AtPP2C2*-*CsPP2C4* | 304.4 | 820.6 | 2.9421 | 0.2638 | 0.0896 |
|  | *AtPP2C3*-*CsPP2C2* | 302.3 | 906.7 | 3.5964 | 0.3502 | 0.0974 |
|  | *AtPP2C14*-*CsPP2C13* | 252.3 | 863.7 | 3.3106 | 0.4108 | 0.1241 |
|  | *AtPP2C9*-*CsPP2C27* | 191.1 | 645.9 | 8.8795 | 0.1424 | 0.0160 |
|  | *AtPP2C7*-*CsPP2C26* | 313.6 | 925.4 | 2.9216 | 0.2720 | 0.0931 |
|  | *AtPP2C16*-*CsPP2C26* | 313.4 | 910.6 | 3.0898 | 0.2528 | 0.0818 |
|  | *AtPP2C15*-*CsPP2C39* | 328.5 | 970.5 | 3.0007 | 0.1390 | 0.0463 |
|  | *AtPP2C4*-*CsPP2C44* | 476.3 | 1470.7 | 3.1900 | 0.1521 | 0.0477 |
|  | *AtPP2C13*-*CsPP2C40* | 269.8 | 828.2 | 5.4349 | 0.3352 | 0.0617 |
|  | *AtPP2C11*-*CsPP2C47* | 185.0 | 640.0 | 3.6382 | 0.1557 | 0.0428 |
|  | *AtPP2C1*-*CsPP2C52* | 358.1 | 1003.9 | 3.3330 | 0.2168 | 0.0651 |
|  | *AtPP2C24*-*CsPP2C2* | 281.2 | 789.8 | 8.4678 | 0.2750 | 0.0325 |
|  | *AtPP2C25*-*CsPP2C4* | 301.6 | 862.4 | 4.8210 | 0.2574 | 0.0534 |
|  | *AtPP2C29*-*CsPP2C16* | 556.9 | 1741.1 | 2.8891 | 0.3154 | 0.1092 |
|  | *AtPP2C23*-*CsPP2C29* | 457.4 | 1441.6 | 52.9892 | 0.3118 | 0.0059 |
|  | *AtPP2C27*-*CsPP2C36* | 283.6 | 853.4 | 2.4396 | 0.1675 | 0.0687 |
|  | *AtPP2C30*-*CsPP2C38* | 294.0 | 753.0 | 45.1570 | 0.4429 | 0.0098 |
|  | *AtPP2C28*-*CsPP2C50* | 205.9 | 616.1 | 5.5087 | 0.3010 | 0.0546 |
|  | *AtPP2C23*-*CsPP2C44* | 433.6 | 1504.4 | 2.9583 | 0.1687 | 0.1687 |
|  | *AtPP2C34*-*CsPP2C9* | 270.3 | 803.7 | 3.8917 | 0.1909 | 0.0491 |
|  | *AtPP2C4*-*CsPP2C9* | 276.0 | 774.0 | 7.3070 | 0.2758 | 0.0377 |
|  | *AtPP2C44*-*CsPP2C11* | 182.3 | 570.7 | 4.6219 | 0.4023 | 0.0870 |
|  | *AtPP2C40*-*CsPP2C19* | 334.9 | 1120.1 | 4.3130 | 0.2265 | 0.0525 |
|  | *AtPP2C35*-*CsPP2C18* | 208.1 | 796.9 | 3.9335 | 0.1888 | 0.0480 |
|  | *AtPP2C46*-*CsPP2C15* | 291.6 | 845.4 | 20.4531 | 0.1712 | 0.0084 |
|  | *AtPP2C48*-*CsPP2C24* | 270.5 | 827.5 | 4.4677 | 0.2080 | 0.0466 |
|  | *AtPP2C39*-*CsPP2C31* | 195.8 | 653.2 | 2.6185 | 0.1771 | 0.0677 |
|  | *AtPP2C36*-*CsPP2C29* | 473.2 | 1404.8 | 7.1241 | 0.4024 | 0.0565 |
|  | *AtPP2C48*-*CsPP2C37* | 272.0 | 868.0 | 3.9175 | 0.1798 | 0.0459 |
|  | *AtPP2C46*-*CsPP2C49* | 267.7 | 869.3 | 2.9576 | 0.1499 | 0.0507 |
|  | *AtPP2C43*-*CsPP2C40* | 269.4 | 825.6 | 7.6269 | 0.3674 | 0.0482 |
|  | *AtPP2C35*-*CsPP2C43* | 211.4 | 832.6 | 4.5279 | 0.1947 | 0.0430 |
|  | *AtPP2C37*-*CsPP2C41* | 262.7 | 817.3 | 5.3276 | 0.4508 | 0.0846 |
|  | *AtPP2C41*-*CsPP2C45* | 257.9 | 774.1 | 1.8185 | 0.3677 | 0.2022 |
|  | *AtPP2C34*-*CsPP2C45* | 261.1 | 785.9 | 7.8743 | 0.3678 | 0.0467 |
|  | *AtPP2C49*-*CsPP2C55* | 302.6 | 840.4 | 16.3447 | 0.3102 | 0.0190 |
|  | *AtPP2C64*-*CsPP2C12* | 313.3 | 877.7 | 2.4676 | 0.1507 | 0.0611 |
|  | *AtPP2C57*-*CsPP2C21* | 312.5 | 842.5 | 5.0522 | 0.2474 | 0.0490 |
|  | *AtPP2C59*-*CsPP2C34* | 208.2 | 667.8 | 1.9644 | 0.0649 | 0.0330 |
|  | *AtPP2C60*-*CsPP2C35* | 239.0 | 829.0 | 2.2789 | 0.1550 | 0.0680 |
|  | *AtPP2C61*-*CsPP2C33* | 226.9 | 712.1 | 9.6373 | 0.4099 | 0.0425 |
|  | *AtPP2C64*-*CsPP2C49* | 309.7 | 884.3 | 3.6036 | 0.1801 | 0.0500 |
|  | *AtPP2C52*-*CsPP2C52* | 347.3 | 1041.7 | 2.4085 | 0.1573 | 0.0653 |
|  | *AtPP2C60*-*CsPP2C56* | 245.4 | 822.6 | 3.0346 | 0.1688 | 0.0556 |
|  | *AtPP2C63*-*CsPP2C54* | 267.4 | 866.6 | 3.0403 | 0.1704 | 0.0560 |
|  | *AtPP2C78*-*CsPP2C2* | 311.8 | 864.2 | 33.0260 | 0.3278 | 0.0099 |
|  | *AtPP2C73*-*CsPP2C5* | 278.2 | 801.8 | 3.4191 | 0.2998 | 0.0877 |
|  | *AtPP2C73*-*CsPP2C9* | 277.5 | 817.5 | 5.3040 | 0.1955 | 0.0369 |
|  | *AtPP2C75*-*CsPP2C20* | 323.9 | 879.1 | 4.2802 | 0.3643 | 0.0851 |
|  | *AtPP2C68*-*CsPP2C25* | 269.0 | 874.0 | 3.7593 | 0.1499 | 0.0399 |
|  | *AtPP2C79*-*CsPP2C15* | 290.8 | 861.2 | 10.6132 | 0.1934 | 0.0182 |
|  | *AtPP2C76*-*CsPP2C22* | 241.8 | 805.2 | 5.5064 | 0.1977 | 0.0359 |
|  | *AtPP2C65*-*CsPP2C30* | 288.5 | 842.5 | 3.4668 | 0.4565 | 0.1317 |
|  | *AtPP2C69*-*CsPP2C34* | 207.8 | 668.2 | 2.6961 | 0.1114 | 0.0413 |
|  | *AtPP2C71*-*CsPP2C34* | 204.1 | 671.9 | 3.9066 | 0.1147 | 0.0294 |
|  | *AtPP2C66*-CsPP*2*C29 | 459.9 | 1475.1 | 5.2639 | 0.4039 | 0.0767 |
|  | *AtPP2C66*-*CsPP2C44* | 456.0 | 1512.0 | 7.8411 | 0.3936 | 0.0502 |
|  | *AtPP2C79*-*CsPP2C49* | 278.9 | 873.1 | 4.0634 | 0.1786 | 0.0440 |
