# Supplementary material for: Genome-wide identification and expression analysis of the cucumber PP2C gene family
Source: BMC Genomics. 2022 Aug 6;23:563. doi: 10.1186/s12864-022-08734-y (PMC9356470; doi:10.1186/s12864-022-08734-y)
Supplement: Supplementary file 2 — Additional file 2: Table S2. Conserved motifs in the amino acid sequences of CsPP2C proteins. [file 12864_2022_8734_MOESM2_ESM.docx]

**Table S2** The sequences of primers used for qRT-PCR.

| **Gene Name** | **Forward Primer Sequence (5'-3')** | **Reverse Primer Sequence(5'-3')** |
| --- | --- | --- |
| PP2C1 | GCAATCTACCCGCAGGAAAGAGC | ATGCCCGAGAAACTTCAAGACGAC |
| PP2C2 | ATGGCTGTTCTCATGTGGCGATG | CGGCGTCTTCCTTCTCATCCTTATC |
| PP2C3 | AGAAGTGGTTGCGAACTGTAGATGC | GGCAGAGAACGGCTTGGAATCG |
| PP2C4 | TGCCTTCGCCGTCCTCGTATAG | AACAGCAGCAGAATACCGATCTTCC |
| PP2C5 | AGAGGCTGAGAGGATAACACAATGC | TCCCATTCGGCAACCAAAGACG |
| PP2C6 | CGAGTTGAGGAACGAGAAGGTTGAG | ATACGCTGGCAGTCAGGTTTGATC |
| PP2C7 | ACAACGACTTCACATCCACCAGTTC | CTCGGCTACAATTAGAGGCGGAATC |
| PP2C8 | GCCGAGCAGTACTATCAAGACAAGG | GTGTAACTCCGAGCAGACCGTTTAG |
| PP2C9 | TGGCTACTGATGGGGTATGGGATG | CGAACCGCACACTCAACCAGAC |
| PP2C10 | TGCAGGATGGAAGCGAAGAAGATG | TCGGCAGGGTTACCAAAGTGTTTAC |
| PP2C11 | TGGCGAGGAATACAAGGAAGAAACG | ATTGATCCACCACCACATGATACCC |
| PP2C12 | GTCAGTGGATGTCATACGGAAGGC | ACACCAACAAGGCAGCAAGATCC |
| PP2C13 | CCGTCAAGGGCAAGAAGAGGTTC | AACAAATGTCGCTGCCTTCCTACC |
| PP2C14 | TTAGTCAGGGCGTCGGTAGTGG | AGTCGGTGCTGCTTCGTTAATGG |
| PP2C15 | CCGTAGGTTCTTGCTGTCTGGTTG | GGACCGAACAAGCCTTCCTAACAC |
| PP2C16 | CTGCTGGTGGTTGTGGTGTTAGAG | AGGAACAGGCTGAAGTGGGAGAG |
| PP2C17 | TGGCGTTGCGGTTTGTTCAGAG | TCTCCTGCACAATCATAGCGTCTTG |
| PP2C18 | CCCATCGTCCTCCACACAAACAC | AAGGCACACGAACAGACTCCAATG |
| PP2C19 | CGAATGCCACCCTAGCGAACAC | AGTAGGCGATGAGGGAACCTTACG |
| PP2C20 | CATCGCCTTCTACCGCTCATCAC | ATTACCGTCGCCGTCCGTTTTC |
| PP2C21 | TCCAAGTCACACTCGGATCAAATGC | TCGCCATGCTGTCTAAGTTCATTCC |
| PP2C22 | CTGGCACTTGGAGAGTAGGAGGAG | CCGCCACAACAAACTGCTTCAAC |
| PP2C23 | GATTCCAACTCTGATCGCCTGCTAC | ATCGCCGTCAATCCCAATTCCAC |
| PP2C24 | GGCGATTCACGAGTAGTGCTTGG | CTCTATGCTTGCGTTGTGTTCTGTG |
| PP2C25 | CGTGTACCAGAGTTATCCGTTCGTG | CAGCCAACAGACCTTCCATTCTCG |
| PP2C26 | CAGTCAGTGGAAGCAGTGGAGATG | AACAACAGCCGTAGAGCCAACAG |
| PP2C27 | AATTCTTGCCAGCGACGGTCTATG | TTGCAGCCTTCAGTGGGTCTTTG |
| PP2C28 | TGCCAATGTTGACTCTGACACTGAG | TGCTTCTGCTGCTAATTGCTTTGC |
| PP2C29 | ACAATGGACCACAGCACTTACACAG | ATAATTGCGGAAGCGTCGTCAGG |
| PP2C30 | GGCGGTTTCCAGGGTCAATGATC | GATGGCTTCTCACGTTCCTCCTTC |
| PP2C31 | GCAAGGGCGTTTGGTGATAGGAG | TGACATGACCTTCCACAATCCATCG |
| PP2C32 | GAAGCGTGAATTGGCGATTGAGAAG | GTTGAAACTCCATCTCCGACCACTC |
| PP2C33 | ATGTCTCCTTCCGCCCTCTCAC | TACTTCGTCGTTGCTCAGCACATC |
| PP2C34 | CAACTGCTTCCACTGCCATCCTAG | CCTCAATTCGCCGTCGCTCATC |
| PP2C3**5** | TTATTCACGCTGGACGAGTCAATGG | TCGCAAAGCTCAACAGTGGTCAC |
| PP2C36 | ACATGAAAGGTGCTAAGGGTTCTGC | GTCTTCCTCCGTCAAGTTCAGTTCC |
| PP2C37 | AGAAGGCATTTCCACGACGACATC | CCGTAGCTTGGACTGCGACTAATC |
| PP2C38 | AAGCAGTTTGTGGCGTAGAGATAGC | CGTCGAGTGAGCCTCTTGATGTTG |
| PP2C39 | CGATGGATGGACGGTAACAGTAGC | TTAAAGCAGAGACAGCACCACCTTG |
| PP2C40 | GGAAGGAATGGGCGGGTTTGTAG | CAGCAATGAGAGGTGATGAGGAGAC |
| PP2C41 | CGTCGAGTGAGCCTCTTGATGTTG | GTGGTGGTGGTGGTGATGATGATG |
| PP2C42 | CGTGTCCTTGTGGATTCAGCTACTC | CATTGGCGAACTCCTGCTCCTTAG |
| PP2C43 | TGTGGGGCTAGAGTTTTGAGTGTTG | ATCGCCGCCTTGACTTTCTTCG |
| PP2C44 | ATGGAGCATTCACTTCCGCTAACC | TTCATCACGGCACCAGCATCATC |
| PP2C45 | GTATGGACTCCTGACTCTGGGACTC | GGTAATGACTCGGTGGCTGACATC |
| PP2C46 | CGGCTGTCGTGTCAGAGTTATGG | CCAGGCTCGCAATGTAGAGTGTC |
| PP2C47 | TCCTCTTCAGTGGTGGCGGTATC | AAGCGACCATCTGCCCATCAAC |
| PP2C48 | GAAGTATGTGACCTGGCTCGAAGAC | GAGGTATTCTGCGGCTGCTTGG |
| PP2C49 | TCACCTCAGAACAGCAGTCAATGTC | ACACCAACCAGGCAGCATGAAC |
| PP2C50 | GGGCAGACTCTAATTGTCGCTCAC | AACAAATCCACCTCGGCTCTCAAC |
| PP2C51 | CCAATGAGTCAGTGAGGCAAGAGC | GCCCTTTACTCGCCAAACATTATGC |
| PP2C52 | ATCTGACTGTGACGAACAAGCCTTC | CCTTGCTTCCTTCGGATGATCTGAC |
| PP2C53 | ACAGGGTGATTTGTGGAGCGAAAC | GAGCGTCGATCAAGATGGCAGTC |
| PP2C54 | AGCGAAGAAGCGGGAAATGAGATAC | GATACACGACGACGACGGTGATG |
| PP2C55 | GGTGGCTATGTTGATGGTGGCTATC | GGTTCTGCAATGAGAGGTGAAGGAG |
| PP2C56 | GTGCCAAGTATCTTCACCGACAGG | TCCACGCATCATCTCATCCATTCTG |
| actin | GCCCTCCCTCATGCCATTCT | TCGGCAGTGGTGGTGAACAT |
